# Multicolour In Vivo Bioluminescence Imaging Using a NanoLuc‐Based BRET Reporter in Combination with Firefly Luciferase

**DOI:** 10.1155/2018/2514796

**Published:** 2018-12-03

**Authors:** Arthur Taylor, Jack Sharkey, Antonius Plagge, Bettina Wilm, Patricia Murray

**Affiliations:** ^1^Department of Cellular and Molecular Physiology, University of Liverpool, Liverpool, UK; ^2^Centre for Preclinical Imaging, University of Liverpool, Liverpool, UK

## Abstract

The ability to track the biodistribution and fate of multiple cell populations administered to rodents has the potential to facilitate the understanding of biological processes in a range of fields including regenerative medicine, oncology, and host/pathogen interactions. Bioluminescence imaging is an important tool for achieving this goal, but current protocols rely on systems that have poor sensitivity or require spectral decomposition. Here, we show that a bioluminescence resonance energy transfer reporter (BRET) based on NanoLuc and LSSmOrange in combination with firefly luciferase enables the unambiguous discrimination of two cell populations *in vivo* with high sensitivity. We insert each of these reporter genes into cells using lentiviral vectors and demonstrate the ability to monitor the cells' biodistribution under a wide range of administration conditions, including the venous or arterial route, and in different tissues including the brain, liver, kidneys, and tumours. Our protocol allows for the imaging of two cell populations in the same imaging session, facilitating the overlay of the signals and the identification of anatomical positions where they colocalise. Finally, we provide a method for postmortem confirmation of the presence of each cell population in excised organs.

## 1. Introduction

Monitoring the biodistribution and fate of cells that are exogenously administered into a host is fundamental for understanding processes in a wide range of biomedical research areas. This includes, for example, understanding the mechanisms of stem cell therapies [1], tumour biology and therapeutic response [2], or host/pathogen interactions [3]. Bioluminescence imaging (BLI) has emerged as an invaluable tool which allows to address such questions in a longitudinal and noninvasive manner. Researchers are now able to easily insert luciferase genes into the cells of interest, administer them to animal models, and immediately identify not only the organs that they populate but also their viability, proliferation, and any changes in biodistribution that might occur over time. When mammalian cells are considered, an enzyme-substrate pair system is usually employed, where the cells of interest are genetically modified to express an enzyme that oxidises a consumable substrate. A product of this reaction is light [4], which can then be captured by a charge-coupled device camera. Of the available systems, the most commonly used is the luciferase from firefly (*Photinus pyralis*) [4] in combination with the substrate luciferin.

The complexity of biological processes, however, will often require that more than one cell population is imaged at the same time to fully address certain scientific questions. For example, it is now recognised that the immune system might play an important role in areas such as stem cell therapies [5] and oncology [6]. In such situations, it might be desirable to monitor not only the stem or cancer cells but also immune-relevant cells such as macrophages or T-cells. Likewise, interactions between host and pathogen would also be better understood by imaging multiple cell populations. Furthermore, the means to monitor multiple cell populations *in vivo* can also reduce animal use in research (3Rs principle), where cells preconditioned in different ways could be coadministered to the same animal and monitored individually.

Attempts to develop systems to monitor more than one cell population in the same animal have often focused on the use of luciferases with different light emission peaks. Dual imaging with a red-shifted firefly luciferase mutant in combination with click beetle luciferase has been reported [7] and so has a combination of red and green luciferases from click beetle [8]. The disadvantage of these approaches is that the light emission spectra of two populations tend to partially overlap, and both luciferases oxidise the same substrate, meaning the light from the two cell populations is emitted at the same time, requiring a spectral decomposition algorithm to clearly identify the anatomical source of each cell population. Spectral unmixing can lead to ambiguities, particularly when *in vivo* imaging is considered, where light attenuation by tissues can significantly affect the spectral properties of luciferase (this is shown for firefly luciferase in Figure S1(a)).

A different approach to imaging more than one cell population is to use luciferases with different substrates, which allows the signal from each cell population to be acquired in sequence, as opposed to simultaneously, thus precluding the need for data acquisition with different emission filters and the postacquisition processing for spectral decomposition. Marine organisms like *Gaussia princeps* and *Renilla reniformis* use coelenterazine as the substrate, but a difficulty associated with luciferases from these organisms is that light emission peaks in the blue/green region of the spectrum [4]. Light in this region is strongly attenuated *in vivo* due to tissue absorption and scattering and results in poor imaging sensitivity, although *in vivo* imaging with those pairs has been reported [9, 10]. More recently, an enzyme called Nanoluc (NLuc), which is a structurally optimised version of the luciferase from the deep sea shrimp *Oplophorus gracilirostris,* has been developed and reported to have brightness superior to that of firefly or renilla luciferases, but with peak emission at 460 nm (blue) [11], thus suffering from the same drawbacks as other marine luciferases. Bioluminescence resonance energy transfer (BRET), where the bioluminescence from a luciferase is used to excite a fluorescent protein, is a natural effect occurring in organisms such as the jellyfish *Aequorea victoria* and now used as a tool to study protein interactions [12]. This principle has been used to build NLuc constructs which are optimised for *in vivo* imaging by fusing this luciferase with a fluorescent protein (FP) with a large stokes shift. In these, NLuc is an energy (light) donor for the fluorescent acceptor, which then emits light >600 nm, an effect that is possible because of the overlap between the light emission peak of NLuc and the absorption peak of the FP. Two of these systems have been recently reported, employing (i) a cyan-excitable, orange-red FP [13] or (ii) a large stokes shift monomeric orange FP [14] (LSSmOrange, spectra shown in Figure S1(b)) as the receptor. Although the performance of the latter for cell tracking has been evaluated *in vivo* [14], no attempts have yet been made to combine either reporter with firefly luciferase in order to establish protocols for the coimaging of multiple cell populations.

Here, we propose to establish and validate the methods for the use of the BRET reporter system LSSmOrange/NLuc in combination with firefly luciferase for monitoring the biodistribution and fate of multiple cell populations *in vivo* and postmortem.

## 2. Materials and Methods

### 2.1. Construct Preparation, Cell Line Transduction, and *In Vitro* Imaging

The plasmids pHIV-Luc-ZsGreen and pRetroX-Tight-MCS_PGK-OgNLuc were gifts from Bryan Welm and Antonio Amelio, respectively (Addgene plasmids #39196, #70186). The pHIV plasmid is a lentiviral construct for bicistronic expression of the codon-optimised firefly luciferase (luc2) and ZsGreen (via an IRES link) under the constitutive EF1*α* promoter. The pRetroX-Tight-MCS_PGK-OgNLuc vector is gamma retroviral and contains the LSSmO_NLuc construct under a PGK promoter. To enable a consistent comparison between firefly luciferase (FLuc) and LSSmO_NLuc, the latter was cloned into a plasmid with the same backbone as pHIV-Luc-ZsGreen, thus resulting in a lentiviral vector with expression of LSSmO_NLuc downstream of EF1*α* promoter (pHIV_OgNLuc). Lentiviral particles were produced by cotransfection of the transfer plasmids with a packaging plasmid (psPAX 2, addgene #12260) and the envelope plasmid (pMD.2G, Addgene #12259) in HEK 293T-cells, followed by concentration by ultracentrifugation and titration via flow cytometry using HEK cells and previously described methods [15].

The mouse mesenchymal stem/stromal cell (MSC) D1 line was obtained from the American Type Culture Collection (CRL-12424), and RAW 264.7 mouse macrophages were obtained from the European Collection of Authenticated Cell Cultures (#91062702). MSC D1s were transduced with the FLuc or LSSmO_NLuc lentiviral particles using a multiplicity of infection (MOI) of ∼5 and polybrene (8 *µ*g/mL in culture medium) and then expanded. RAW cells were transduced with FLuc particles without polybrene, which is toxic to this line, and the positive cell fraction was then sorted based on ZsGreen expression using an Aria fluorescence-activated cell sorter (BD Biosciences). In all cases, cells were maintained in Dulbecco's Modified Eagle's medium with 10% foetal calf serum at 37°C in a humidified incubator with 5% CO_2_.

For *in vitro* imaging, cells were suspended in polypropylene tubes or plated at different densities in black 96-well plates and allowed to settle for 2–4 h. Then, D-luciferin (150 *µ*g/mL) or furimazine (20 *µ*g/mL), both from Promega, were added to the tubes or wells immediately prior to imaging with an IVIS spectrum system (PerkinElmer). These are working concentrations for *in vitro* studies as suggested by PerkinElmer for D-luciferin and Promega for furimazine. All bioluminescence data have been normalised to the acquisition conditions and are displayed in radiance (photons/s/cm^2^/str). To obtain the bioluminescence spectra of the cells, data were acquired with each of the emission filters available in the system (500 to 800 nm in 20 nm steps), and the normalised signal was plotted as a function of wavelength. For quantification of light output, ROIs were drawn around each of the tubes/wells, and the flux in photons per second (p/s) was calculated using Living Image (PerkinElmer). A background control consisting of medium and substrate only was included in all *in vitro* experiments and subtracted from the experimental values.

### 2.2. *In Vivo* Imaging

BALB/c mice were purchased from Charles River and housed in individually ventilated cages under a 12 h light/dark cycle, with *ad libitum* access to standard food and water. All animal experiments were performed under a license granted under the UK Animals (Scientific Procedures) Act 1986 and were approved by the University of Liverpool ethics committee. All procedures (administration of cells/substrates and imaging) were carried out under anaesthesia with isoflurane.

For administration into animals, cells were harvested, counted, suspended in phosphate buffered saline (PBS), and kept on ice until injection. Cells (MSCs or a combination of MSCs and RAWs) were suspended in 100 *µ*L for injection via the tail vein or into the arterial system via the left cardiac ventricle as previously described [16].

For imaging, furimazine was used at a dose of 250 *µ*g/kg body weight and luciferin at a dose of 150 mg/kg body weight. For the initial optimisation of the administration route, mice received MSCs intravenously (IV) and were then imaged on the same day immediately after administration of furimazine subcutaneously (SC), intraperitoneally (IP), or IV via a cannula. In all subsequent experiments, the mice were cannulated, moved to the imaging chamber, and then the substrates were administered IV immediately prior to data acquisition. The total flux from mice was quantified in the same manner as described above for *in vitro* imaging. Changes in signal intensity over time were captured with sequential acquisition of data with intervals of 1 or 2 min.

For sequential data acquisition from mice that received cells expressing both reporters, animals were moved to the imaging chamber and injected with furimazine, luciferin, or PBS at different time points. For animal welfare reasons, we aimed at keeping the injection volumes as low as possible (i.e., a total administration volume ≤200 *µ*L). To overlay the images obtained with each of the reporters, the raw luminescence images are displayed with pseudocolours and overlaid with the photograph using ImageJ.

## 3. Results

### 3.1. The NLuc BRET Reporter is Effective and Yields a Light Output that Exceeds Firefly Luciferase

**Figure 1:**
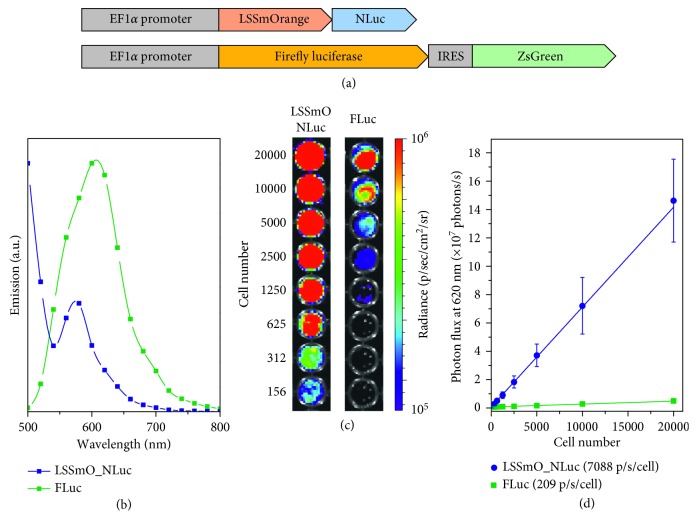
Bioluminescence of MSCs expressing a FLuc or an LSSmO_NLuc construct. (a) Arrangement of the lentiviral inserts used to transduce the cells (not to scale). (b) Bioluminescence spectra of MSCs transduced with each of the constructs. (c) Bioluminescence imaging of different numbers of MSCs expressing LSSmO_NLuc (left) or FLuc (right). (d) Light output as a function of cell number. Error bars denote SD from *n*=3 experiments. Luciferin (150 *µ*g/mL) was used as a substrate for FLuc and furimazine (20 *µ*g/mL) as a substrate for NLuc. An emission filter (620 nm) was applied.

MSC D1s were transduced with a lentiviral vector for the overexpression of FLuc or LSSmO_NLuc downstream of an EF1*α* promoter (Figure 1) using an MOI of ∼5. In both cases, over 90% of the cells expressed luciferase when assessed via immunofluorescence microscopy (Figure S2). The emission spectrum of cells expressing FLuc consisted of a single peak centred at ∼605 nm (Figure 1). MSCs expressing LSSmO_NLuc, on the contrary, displayed two emission peaks, one at 500 nm, which is likely to correspond to the shoulder of the NLuc peak that is centred at 460 nm, and a second peak at ∼575 nm which corresponds to the emission peak of LSSmO (Figure 1), confirming the functionality of the BRET insert. To compare the performance of each reporter at conditions relevant for *in vivo* imaging, the light output of the cells was measured with a 620 nm emission filter (bandwidth 18.9 nm) to exclude any light that would normally be attenuated by tissues. This condition also excludes any NLuc light that is not transferred to LSSmO. Cells expressing either of the reporters presented significant light emission (Figure 1), but the output of those expressing LSSmO_NLuc was nearly 35-fold higher than that of cells expressing FLuc (7088 vs. 209 p/s/cell), suggesting a significantly greater brightness for this reporter system (Figure 1). Without any emission filters, the measured light output was 204043 p/s/cell for LSSmO_NLuc and 1508 p/s/cell for FLuc.

### 3.2. Route of Furimazine Administration Impacts Specificity of Bioluminescence Signal

**Figure 2:**
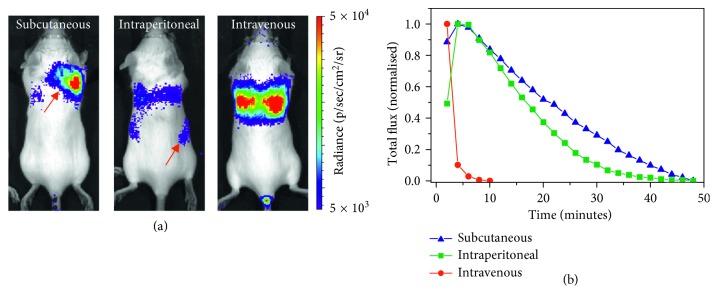
Impact of substrate administration route on signal specificity. MSCs (10^6^) expressing LSSmO_NLuc were administered via the tail vein and, on the same day, the mice were imaged using furimazine as a substrate. (a) Representative BLI of mice that received the substrate SC, IP, or IV. These cells are known to lodge in the lungs. Red arrows indicate areas with nonspecific signal. The signal obtained via each route was probed with at least three individual animals. (b) Representative total flux as a function of time as measured from the whole animal (one acquisition every 2 minutes).

To determine adequate routes of administration for the NLuc substrate (furimazine), mice received 10^6^ MSCs expressing LSSmO_NLuc via the tail vein. Previous data suggest that this route of administration leads to the cells immediately lodging exclusively in the lungs [16]. Administration of furimazine subcutaneously (under the skin of the neck) resulted in a strong signal in this area (Figure 2), which was not specific to the lungs, suggesting that the substrate did not efficiently reach the circulation. It should be noted that furimazine (and other coelenterazine analogues) gives background signal because of light emission from enzyme-independent oxidation (autoluminescence). This is most likely the source of the signal we have observed under this condition. When administered IP, we detected a weak but specific signal originating from the lungs suggesting that some of the substrate was absorbed and reached this organ. However, nonspecific signal was also observed in the peritoneum (Figure 2, red arrows), indicating that significant amounts of the substrate were trapped in this region. The IV route was the only administration route that yielded a strong, specific signal originating only from the lungs. The substrate clearance, as measured by imaging the mice for up to 50 minutes post-administration of furimazine, was also distinct for each of those routes. Whereas the luminescent signal persisted for more than 30 minutes when the substrate was given SC or IP, the signal cleared very quickly after IV administration, and was lost within 10 minutes of injection (Figure 2). This is in contrast to luciferin, which has a longer half-life (Figure S3).

### 3.3. FLuc Signal *In Vivo* Is Stronger Than LSSmO_NLuc

To assess whether the differences in signal intensity observed *in vitro* are reflected *in vivo*, mice were injected with MSCs expressing either of the reporters via the tail vein and imaged for up to 3 days. For a direct comparison of light output, animals received the substrate IV irrespective of the reporter system and were imaged immediately after administration under identical acquisition conditions. The substrate was administered via a cannula as the animal was in the imaging chamber, enabling data acquisition to start within 5 seconds of administration.

**Figure 3:**
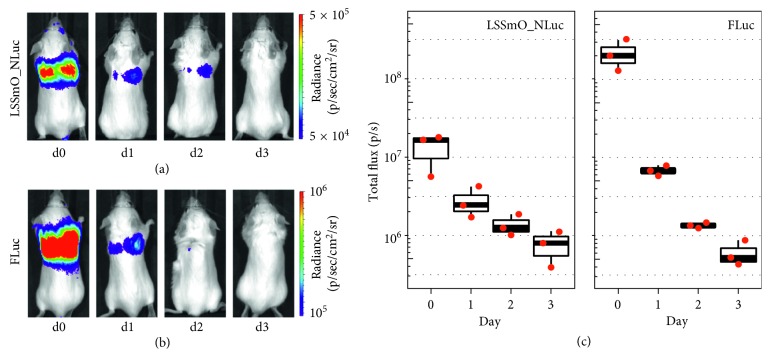
Bioluminescence imaging of mice that received 10^6^ MSCs expressing (a) LSSmO_NLuc or (b) FLuc. The colour intensity scale has been adjusted for each reporter system. (c) Quantification of the signal in the lungs on each of the measurement days (*n*=3). Imaging conditions were identical for both reporter systems: medium binning, f/stop 1, exposure time 45 s.

For both systems, an intense, specific signal originating from the lungs was seen on the administration day (d0) which gradually decreased on the subsequent days suggesting cell death (Figures 3 and 3). The images for all time points are displayed in the same colour scale to allow a direct comparison of the signal changes over time, but it should be noted that some signal was still present in the lungs by day 3 (shown in Figure S4). Interestingly, the signal intensity observed for the LSSmO_NLuc construct was not stronger than that of FLuc, and in fact, it was weaker on days 0 and 1. This is reflected in the quantification of the total flux (Figure 3), where the mean FLuc signal was 15-fold stronger than LSSmO_NLuc on the administration day (d0), 2.5-fold stronger on day 1, and not different or weaker than LSSmO_NLuc on days 2 and 3.

### 3.4. Fast Furimazine Clearance Enables Tracking of Two Different Cell Populations Individually under the Same Imaging Session

Having validated the sensitivity of both reporters, we sought to evaluate whether it is possible to track two distinct cell populations independently. For this purpose, we chose to coinject MSCs expressing LSSmO_NLuc and RAW macrophages expressing FLuc. We injected both cell types simultaneously via the left cardiac ventricle (10^6^ each) as our previous studies have shown that this is the most efficient way to achieve a whole body distribution of cells [16]. This approach enables cells to populate a variety of organs, instead of being trapped in the lungs.

We explored the very short half-life of furimazine for coimaging the two different cell types. Because the signal is cleared so quickly, it is possible to image both populations in the same session as long as the signal from furimazine expressing cells is acquired first. We established an imaging protocol that consisted of (1) tail vein cannulation, (2) administration of furimazine IV and immediate data acquisition to obtain the LSSmO_NLuc signal, (3) a waiting period of 15–20 minutes to allow furimazine to clear, where the cannula is flushed to wash away any remaining furimazine, and (4) administration of luciferin IV and immediate data acquisition to obtain the FLuc signal.

**Figure 4:**
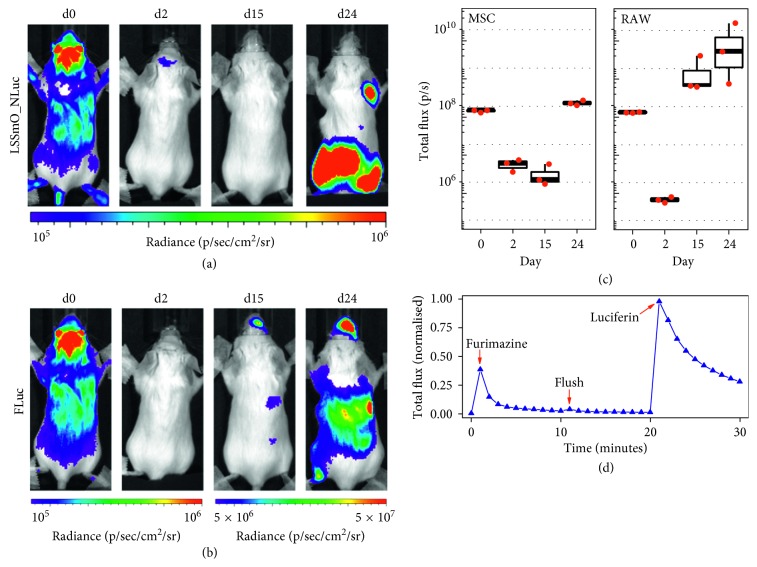
Coimaging of mice that received MSCs expressing LSSmO_NLuc and RAWs expressing FLuc (10^6^ of each cell). (a) The MSC BLI as obtained from LSSmO_NLuc at different time points. (b) The RAW BLI as obtained from the FLuc reporter at different time points. (c) Quantification of the BLI originating from the whole body of the mouse on each of the measurement days (*n*=3). Imaging conditions: medium binning, f/stop 1, 50 s exposure time for all images except for the acquisition of RAW data on days 15 and 24, where shorter exposure times were applied to avoid saturation of the detector. (d) Typical signal intensity evolution as a function of time as measured from the whole animal (one acquisition every 1 minute). The chart shows the changes in signal as the different substrates are administered to a mouse via a cannula.

The BLI signal of MSCs and RAWs revealed whole body distribution of both cell types on the administration day, with signal clearly originating from the brain, liver, lungs, and kidney (Figures 4 and 4). This distribution is in agreement with cell administration for both cell types via the arterial route. BLI on the following days demonstrated this is followed by a period where almost no signal was detected, and then, a fast increase in signal intensity, suggesting proliferation of both cell types (Figures 4 and 4). MSCs proliferated mainly close to the limbs, particularly the hindlimbs, whereas the RAWs proliferated in the head and in the abdominal region. These observations are supported by quantification of the mean total flux from a ROI covering the whole animal's body (Figure 4), showing that the MSC signal only starts to increase 15 days after administration, whereas the RAW signal increases earlier, between days 2 and 15.

MRI analysis of the mice on day 24 revealed the presence of tumours in the skeletal muscle, particularly in the hind legs and spine, and also in or around the liver (Figure S5), in good agreement with the anatomical sites where bioluminescence was observed. A chart displaying the typical signal flux from such experiments is shown in Figure 4. There is a peak in signal intensity immediately after furimazine administration, when the MSC data are acquired, followed by quick decay in signal, until the syringe is flushed and a minimal increase is seen as a response to the substrate left in the cannula. After a period of 15–20 minutes, the signal is completely cleared and luciferin is injected for acquisition of the FLuc data.

**Figure 5:**
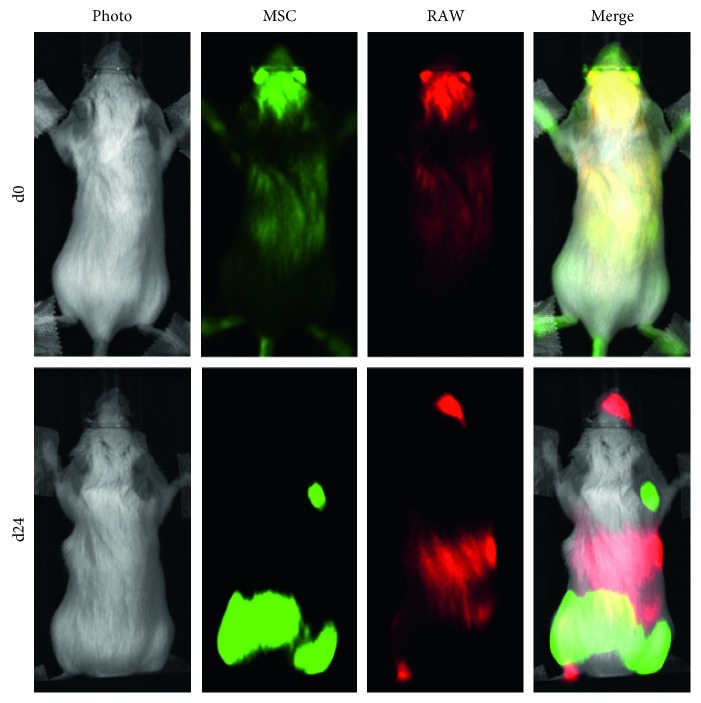
Identifying colocalisation of MSCs and RAWs *in vivo*. The bioluminescence signal from each of the exogenously administered cell types is displayed in pseudocolours (green, MSCs; red, RAWs) and then overlaid with a photograph of the mouse. Whereas a strong co-localisation of the cells is seen on day 0, resulting in a yellow colour when the channels are merged, the two different populations had proliferated at different anatomical locations by day 24, with minimal co-localisation at this time point.

Data acquisition in a single imaging session allowed us to overlay the signal originating from each of the cell populations. The signal from the MSCs and RAWs on day 0 or 24 is shown in pseudocolours, together with an overlay of the images, in Figure 5 and corresponds to the same data as shown in Figures 4 and 4. This approach demonstrates that, on the administration day (d0), the signals from MSCs and RAWs strongly colocalised throughout the whole body, whereas on day 24, the cells were present and had proliferated at different anatomical sites.

### 3.5. Identification of Distinct Cell Population Postmortem

**Figure 6:**
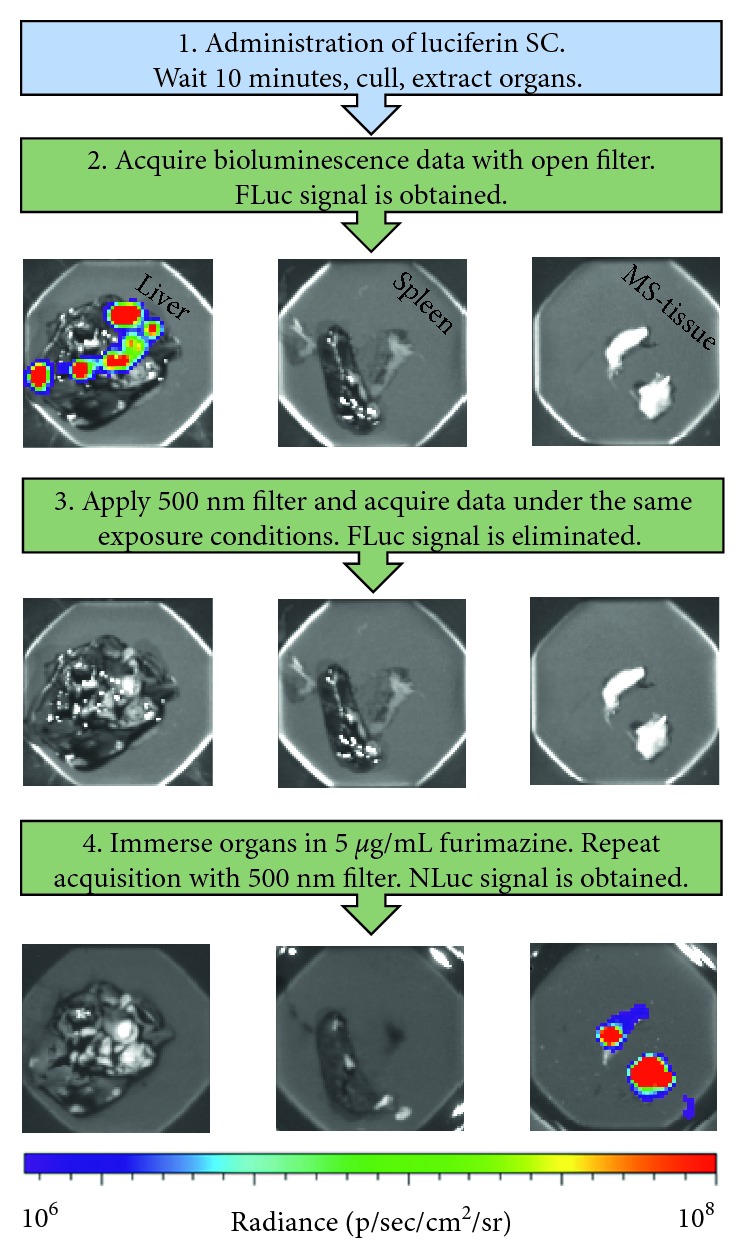
Identification of each cell population in organs postmortem. The liver, the spleen, and tumour-containing musculoskeletal (MS) tissue samples were harvested from mice and imaged with a protocol that allows the identification of FLuc (shown in step 2) or LSSmO_NLuc cells (shown in step 4). Here, livers contained foci of RAW cells expressing FLuc, the spleen contained no exogenously administered cells, and the tumour-containing musculoskeletal tissue consisted of MSCs expressing LSSmO_NLuc. The radiance scale is the same for all images.

In order to establish a protocol to identify the two different cell populations postmortem, we selected the liver, the spleen, and tumour-containing musculoskeletal tissue from the mice on day 24 as exemplary organs where we suspected the MSCs and/or RAWs had populated. We opted for the development of a protocol with sequential data acquisition, thus providing unambiguous signal data from each of the reporters. In this case, however, the order of the data acquisition is inverted and the FLuc signal is acquired before LSSmO_NLuc to take advantage of the longer luciferin half-life. First, the mice received a SC injection of luciferin 10 minutes prior to cervical dislocation. It is well known that this allows the substrate to reach the circulation and all organs. Then, after confirmation of death, the tissues were harvested, collected in black weighing boats, and imaged with no filters applied. These processes capture the total luminescence from tissues that contain FLuc (RAW cells).  Figure 6, Step 2 displays the data obtained under these conditions, which revealed signals in the form of foci in the livers, which is consistent with the pattern seen via MRI. No RAW cells were present in the spleen or in the musculoskeletal tissue samples. Subsequently, a 500 nm (bandwidth 20.2 nm) filter is applied, which represents the shortest available wavelength in the IVIS spectrum system. Applying this filter eliminates any signal originating from FLuc (Figure 6, Step 3). Finally, the containers with the tissues are filled with furimazine (5 *µ*g/mL in PBS), allowed to settle for 1–2 minutes and then imaged as in the previous Step (500 nm filter and the same exposure conditions). This enables the camera to capture any NLuc photons that are not transferred to LSSmO and is thus specific to areas containing the MSCs. Here, we observed that no signal originated from the livers or spleen, but a strong, specific, signal originated from musculoskeletal tissue samples containing tumours.

## 4. Discussion

We have aimed at establishing a bioluminescence imaging strategy to unambiguously monitor the biodistribution of two cell populations independently. We opted to utilise FLuc expression in one of the cell populations, given its widespread adoption, whereas for the second cell population, we opted to use a BRET reporter that uses the substrate furimazine. To allow direct comparison of these systems, we used the same constitutive promoter and equivalent MOI so that the gene expression levels of the two reporters in the MSCs would be similar. When measured in a dish, cells expressing LSSmO_NLuc displayed a much greater signal than those expressing FLuc. Although the use of the same promoter and MOI does not guarantee the same expression levels, the differences observed are too great to be explained only by different levels of protein expression between the cells. Given the well-documented brightness of NLuc, we suggest that our results reflect the brightness of this luciferase and an efficient energy transfer to LSSmO.

When the two luciferase systems were compared *in vivo*, however, the difference in brightness was not observed. This finding could be based on the poor bioavailability of furimazine which, unlike luciferin, has poor water solubility and is commercialised as a stock solution in ethanol. In fact, we have noticed the formation of precipitates if furimazine is diluted in PBS and kept on ice for long periods (>30 min). In the experiments reported here, the substrate was always diluted just before administration to prevent precipitation, as this maximises the signal output. However, this step was not enough to ensure efficient delivery to the circulation when using the SC or IP routes. It is interesting that the original research paper describing the LSSmO_NLuc construct involves several experiments in which furimazine is administered IP [14] without the authors giving any indications of unspecific background. However, the authors moved to IV injections towards the end of the study, suggesting that they also saw advantages using this route. An earlier study involving the imaging of NLuc expressing cells also reported a significant background signal when IP administering furimazine [17], suggesting that this is a common issue. However, even though IV administration seems to be the only suitable route for this substrate, its half-life is very short, indicating poor bioavailability and suggesting the need for improved, aqueous soluble substrates, if signal output with this reporter is to be maximised. Indeed, our kinetic measurements suggest a half-life much shorter than 1 minute (the temporal resolution used here). This probably impacts the flux measurement, as in our experiments we fixed the acquisition time to 45–50 s. Because substrate availability to the enzyme is unlikely to be that long, shorter acquisition times might lead to a higher flux. Yet, even with these shortcomings, the signal intensity was strong enough for easy identification of the cell's anatomical location after IV or IC administration, confirming the suitability of both reporter systems for tracking cell biodistribution. Importantly, LSSmO_NLuc was no less sensitive than FLuc to detect the small number of cells that remained in the lungs of mice 3 days after IV administration, a period after which the majority of the administered cells have already died.

We have used the short half-life of furimazine to our advantage, as it allowed us to establish a protocol for imaging the two cell populations under the same data acquisition session. One of the major advantages of this method is that the animal is not moved between imaging of each reporter, facilitating the overlaying of the data. We noticed that this is further improved by gently taping the limbs of the animal to the chamber, preventing any movement that might affect the quality of the data. Our imaging protocol provides clear, unambiguous acquisition of the signals from each luciferase individually, and the different conditions used here (delivery to the lungs, whole body delivery, and tumour formation in different organs) suggest that this protocol is robust enough to allow imaging under a range of conditions and at different tissue depths with success. This method is also appealing for facilities that have imaging systems without emission filters, a requirement for methods that rely on spectral decomposition. It should be noted that, although we have been able to image cells delivered to the brain via the arterial system, these cells are probably trapped in brain capillaries and have not reached the parenchyma [16]. The imaging of cells expressing LSSmO_NLuc in the parenchyma of a brain with an intact blood-brain barrier (BBB) still requires validation, as some substrates can have limited BBB permeability.

When the long-term behaviour of MSCs and RAWs are considered, both cell populations formed tumours, but in different organs and at different rates. Our previous data showed that these MSCs tend to die within the first days of administration but a small pool of cells survive and form osteosarcomas [16]. This is likely a consequence of their extensive expansion *in vitro* leading to malignant transformation, a well-documented effect [18]. The RAW macrophages, on the contrary, are known to be tumourigenic [19]. In our study, these cells underwent significant cell death in the first days but formed tumours in the livers by the end of the experimental period. Thus, while the longitudinal behaviour of these two cell types was not unexpected, their unique *in vivo* fates facilitated the validation of the efficacy of the reporter systems under different conditions of cell distribution and tumour formation.

Because spatial resolution of BLI is poor when compared with other imaging modalities, it is often desirable to confirm the location of the cells of interest by imaging organs *ex vivo* at the experimental endpoint. This is also the case for situations where only small numbers of viable cells are left in the tissues, preventing *in vivo* detection due to poor sensitivity. Thus, we established a protocol with sequential data acquisition that involved the detection of FLuc at 605 nm and the detection of LSSmO_NLuc at 500 nm (note that the BRET reporter emits at both 460 nm and 570 nm as the energy transfer is not 100% efficient). With this method, we were able to unambiguously detect each of the cell populations in different organs. Although we were imaging tumours that were large enough to be detected by MRI, we anticipate that the sensitivity of this method would permit visualisation of small numbers of cells, particularly for the population expressing NLuc, as this luciferase yields very strong signal intensities *ex vivo*, where there is less tissue attenuation. We opted to leave the organs intact, but mincing them in smaller parts is likely to further increase detection sensitivity of NLuc, by enabling a more efficient penetration of the substrate into the tissues.

Finally, it should be noted that the use of these two luciferase systems could be further expanded for other purposes. For instance, there is potential for tracking up to three different cell populations by combining with firefly luciferases that have different emission spectra, although this would entail the drawbacks associated with spectral decomposition. This could be justified depending on the experimental design and the research questions being addressed. There is also a drive for imaging gene expression *in vivo* by using constructs where luciferase expression is driven by promoter activity [20], enabling specific milestones to be identified *in situ* such as the differentiation of stem cells postadministration or the activation of metastatic genes in cancers. In such cases, the combination of these reporters in the same cell would enable a very sensitive detection of gene expression (for example, using FLuc downstream of specific promoters) and, at the same time, the monitoring of cell proliferation and/or viability (using LSSmO_NLuc downstream of a constitutive promoter).

## 5. Conclusions

Distinct cell populations expressing FLuc or the BRET reporter LSSmO_NLuc can be unambiguously discriminated *in vivo* with similar sensitivities under a wide range of conditions including cells distributed throughout the whole body or in the form of tumours in specific organs. Although the poor bioavailability of furimazine requires intravenous administration of this substrate, its short half-life enables the imaging of both populations in the same session, facilitating image overlay and the identification of areas of colocalisation. With the application of light emission filters, this imaging system allows the postmortem confirmation of tissues that have been populated by each cell fraction.

## Data Availability

The data used to support the findings of this study are included within the article and the supplementary information files.

## Supplementary Materials

Supplementary Materials Figure S1: spectra of the reporter systems. Figure S2: immunostaining of MSCs expressing FLuc or LSSmO_NLuc. Figure S3: the time-dependent total flux from mice that received MSCs expressing LSSmO_NLuc or FLuc, after IV administration of the respective substrate (furimazine or luciferin). Figure S4: data from Figure 3 as displayed at a lower signal intensity scale. Figure S5: MR imaging reveals the formation of multiple tumours after MSC and RAW injection into BALB/c mice.
