# Supplementary material for: Multicolour In Vivo Bioluminescence Imaging Using a NanoLuc‐Based BRET Reporter in Combination with Firefly Luciferase
Source: Contrast Media Mol Imaging. 2018 Dec 3;2018:2514796. doi: 10.1155/2018/2514796 (PMC6305057; doi:10.1155/2018/2514796)
Supplement: Supplementary Materials — Figure S1: spectra of the reporter systems. Figure S2: immunostaining of MSCs expressing FLuc or LSSmO_NLuc. Figure S3: the time-dependent total flux from mice that received MSCs expressing LSSmO_NLuc or FLuc, after IV administration of the respective substrate (furimazine or luciferin). Figure S4: data from Figure 3 as displayed at a lower signal intensity scale. Figure S5: MR imaging reveals the formation of multiple tumours after MSC and RAW injection into BALB/c mice. [file 2514796.f1.pdf]

Contrast Media and Molecular Imaging

Multicolour *in vivo* bioluminescence imaging using a NanoLuc-based BRET reporter in combination with firefly luciferase

Arthur Taylor[1,2], Jack Sharkey[1,2], Antonius Plagge[1,2], Bettina Wilm[1,2], Patricia Murray[1,2]

1. Department of Cellular and Molecular Physiology, University of Liverpool, Liverpool, UK,

2. Centre for Preclinical Imaging, University of Liverpool, Liverpool, UK.

Supplementary Material

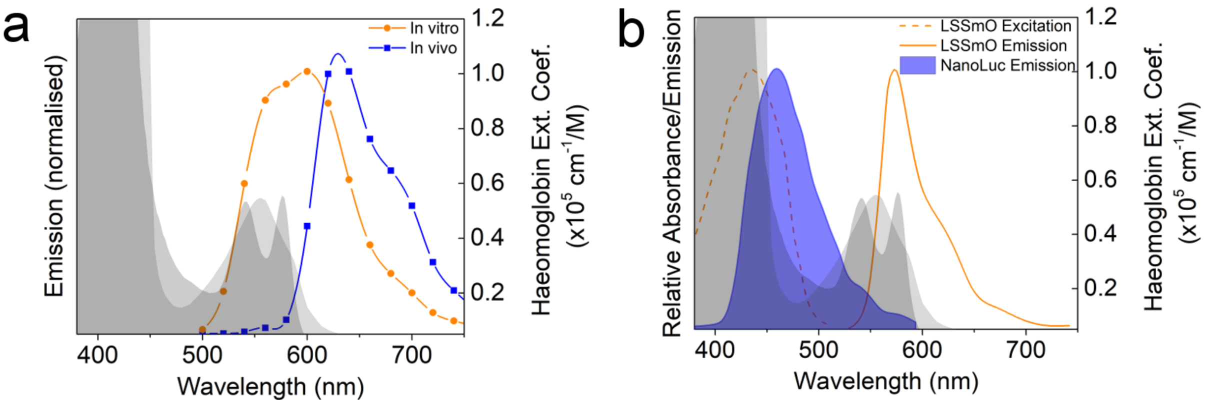

Fig. S1. Spectra of the reporter systems. **(a)** Spectra from a cell line (RAW macrophages) expressing firefly luciferase. Data was acquired *in vitro* (from a culture plate) or *in vivo* (from a mouse after the cells populated the liver). Although the peak emission is at ~ 600 nm when imaged *in vitro*, this is not reflected *in vivo* because of light attenuation by tissues. *In vivo*, only light > 600 nm is detected efficiently. The grey spectra correspond to absorption from oxy- (dark grey) and deoxyhaemoglobin (light grey). **(b)** Emission spectrum of NLuc (reproduced from ref. 11) and the excitation and emission spectra of LSSmOrange (reproduced from doi: 10.1021/ja3018972). The overlap between the emission of NLuc and the excitation of LSSmOrange allows energy transfer and light emission > 600 nm, where haemoglobin attenuation is minimised.

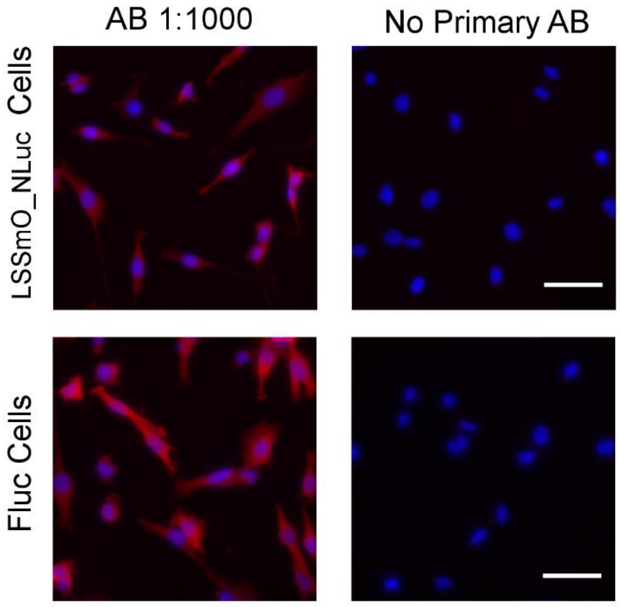

Fig. S2. Immunostaining of MSCs expressing FLuc or LSSmO_NLuc. The cells were plated in culture chambers, fixed, permeabilised with Triton-X 100 and stained with an antibody against NLuc (non-commercial product obtained as a gift from Promega) or FLuc (Abcam, ab21176). Both antibodies were raised in rabbit and a chicken-anti-rabbit Alexa fluor 568 was used as a secondary antibody (Thermo Scientific). The slides were imaged with an epifluorescence microscope and no primary antibody controls were imaged with the same exposure conditions. Scale bar corresponds to 50 µm.

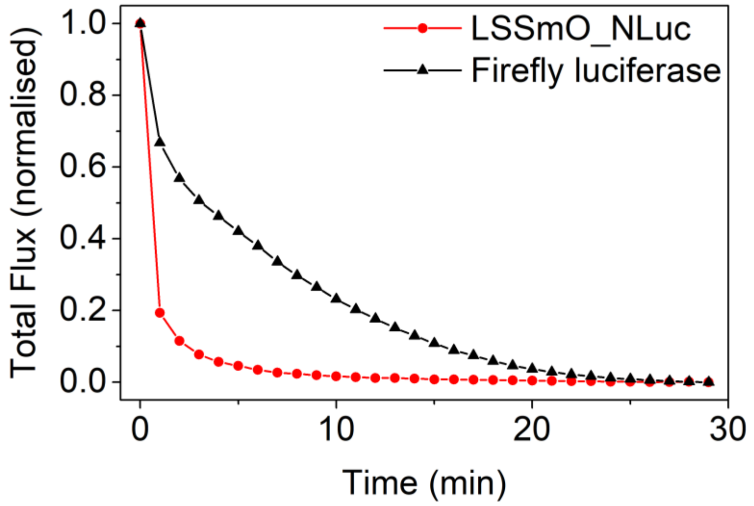

Fig. S3. The time-dependent total flux from mice that received MSCs expressing LSSmO_NLuc or FLuc, after IV administration of the respective substrate (furimazine or luciferin). The changes in total flux provide an indirect measurement of the substrate's bioavailability and suggests that furimazine is rapidly cleared from the circulation, whereas luciferin persists for over 20 minutes.

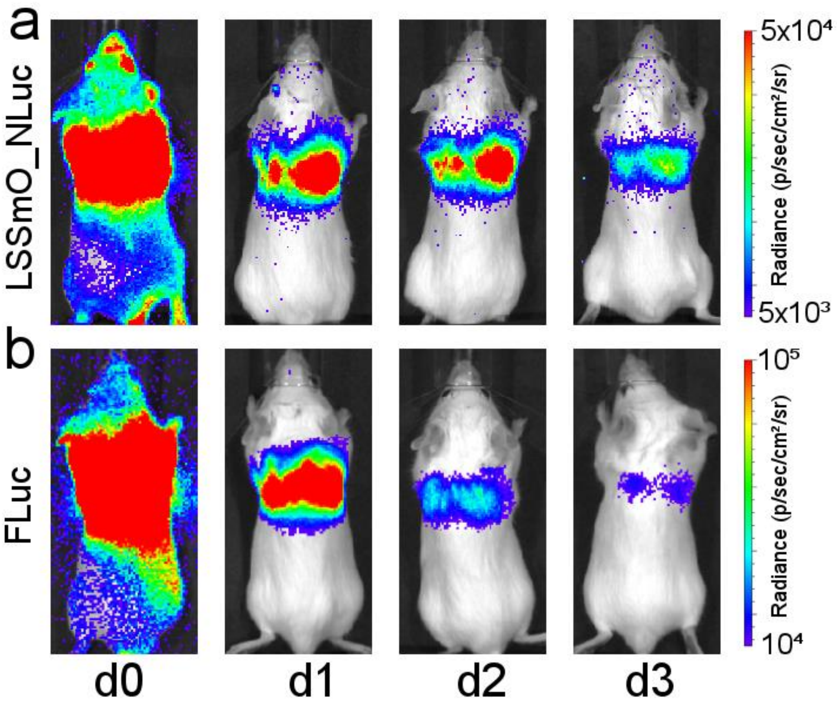

Fig. S4. Data from Figure 3 as displayed at a lower signal intensity scale. (a) MSCs expressing LSSmO_NLuc, (b) MSCs expressing FLuc. There is significantly signal bleed on d0 under this scale, but it enables the visualisation of a weak signal by day 3. Colour intensity scale has been adjusted for each reporter system.

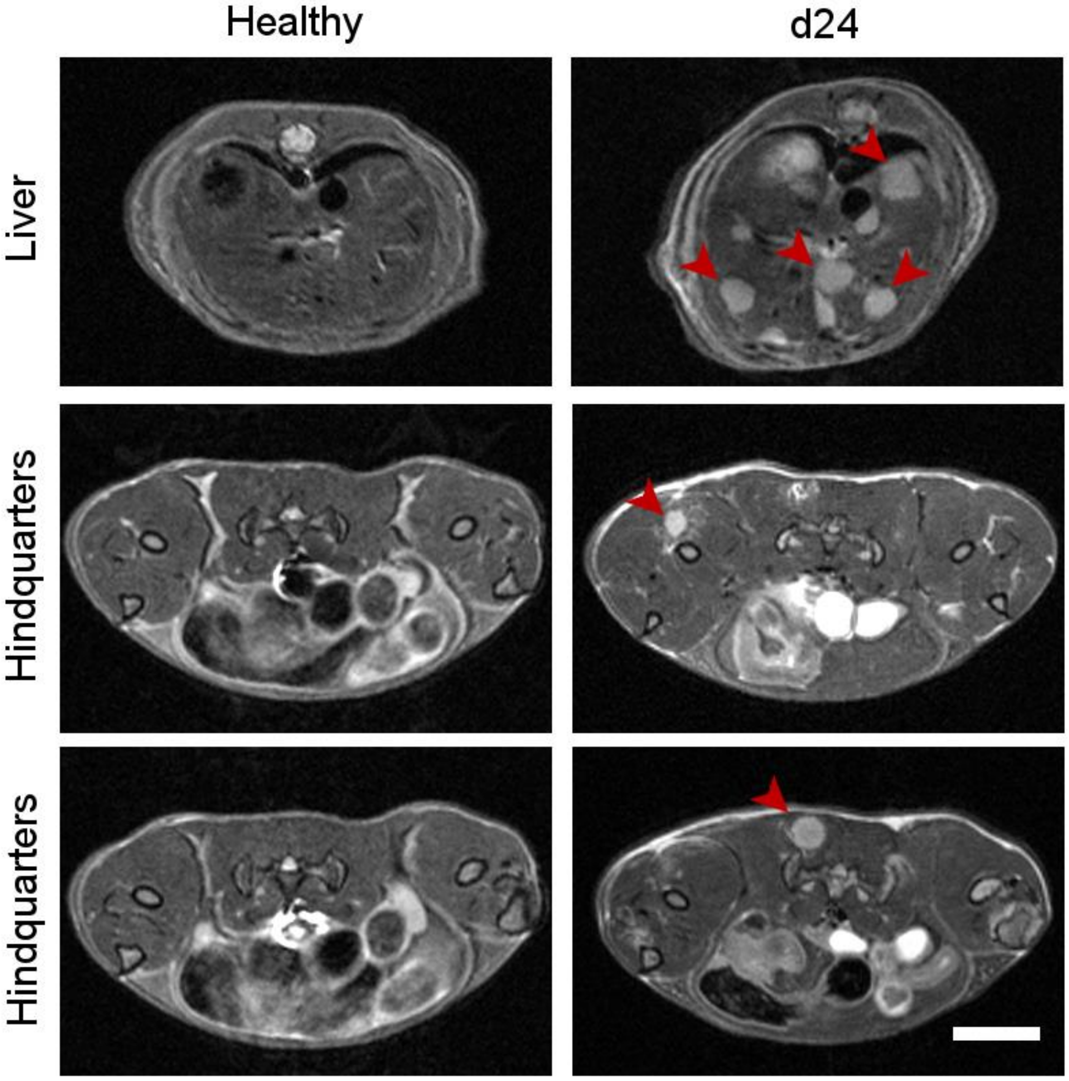

Fig. S5. MR imaging reveals the formation of multiple tumours after MSC and RAW injection into BALB/c mice. In the left panels, axial images of the liver or hindquarters of a healthy animal are shown. In the right panels, images of similar regions from a mouse 24 days post administration of MSCs and RAWs (10^6 each) reveal multiple tumours seen as hyperintense nodules in the liver and close to bones (red arrowheads). MR data was acquired with a Bruker Avance III spectrometer interfaced to a 9.4T magnet system (Bruker Biospec 90/20 USR) using a 40 mm transmit/receive volume coil. Mice were imaged with a T_2 weighted RARE sequence with the following parameters: TE: 25 ms, TR 2500: ms, FOV: 40x30 mm, matrix 256x192 pixels, slice thickness: 0.5 mm, number of slices: 30, NEX: 5, scan time: 5 minutes
